# Surgery

**Published:** 1863-05

**Authors:** 


### SURGERY.

It is proposed to devote a few pages to a description of several surgical maladies, such as are very commonly met with in every community, and which, from the fact of almost total ignorance of them on the part of the people as to their nature and beginnings, often assume a fatal form before either patient or friend has the most remote idea of danger. Men die of heart-disease, when such an event might have been indefinitely postponed had its existence been early discovered by the skillful surgeon; so with various forms of tumors, ruptures, aneurisms, etc., etc. Among the maladies described in the following pages are —

TUMORS—permanent and transient, internal and external.

FACE—its various deformities, congenital or accidental, modes of removing, etc.

MOTHER'S MARK—nature, remedy, etc.

BURNS on neck, cheek, eye, etc., and remedy.

FISTULA of the eye, rectum, etc. — their nature, cause, cure, and remedy.

EYE — its deformities — squinting, watery eyes, or fistula lachrymalis.

NOSE—congenital and accidental disfigurements, its restoration, etc.

ANAL AND RECTAL diseases, fistula, etc.

PILES — their cause, nature and permanent cure, whether bleeding or blind, external or internal.

WARTS, excrescences, etc.

KIDNEYS—diabetes, strictures, debilities, runnings, sores, etc.

